# Two-step catalytic conversion of lignocellulose to alkanes†

**DOI:** 10.1039/c9ra03174j

**Published:** 2019-07-30

**Authors:** Zhuohua Sun, Daniel Buwalda, Katalin Barta

**Affiliations:** Stratingh Institute for Chemistry, University of Groningen Nijenborgh 4 9747 AG Groningen The Netherlands k.barta@rug.nl sunzhuohua@yahoo.com

## Abstract

Direct conversion of lignocellulose to alkanes is challenged by the complex and recalcitrant nature of the starting material. Generally, alkanes are obtained from one of the main lignocellulose constituents (cellulose, hemicellulose or lignin) after their separation, and platform chemicals derived therein. Here we describe a two-step methodology, which uses unprocessed lignocellulose directly, targeting a mixture of alkanes. The first step involves the near-complete conversion of lignocellulose to alcohols, using a copper doped porous metal oxide (Cu-PMO) catalyst in supercritical methanol. The second step comprises a novel solvent exchange procedure and the exhaustive hydrodeoxygenation (HDO) of the complex mixture of aliphatic alcohols, obtained upon depolymerization, to C_2_–C_10_ alkanes by either HZSM-5 or Nafion at 180 °C in conjunction with Pd/C in dodecane. This describes an unprecedented two-step process from lignocellulose to hydrocarbons, with an overall carbon yield of 50%.

## Abstract 2

This work described a simple two-step process for the complete lignocellulose conversion to alkanes with high carbon yield.

## Graphical abstract

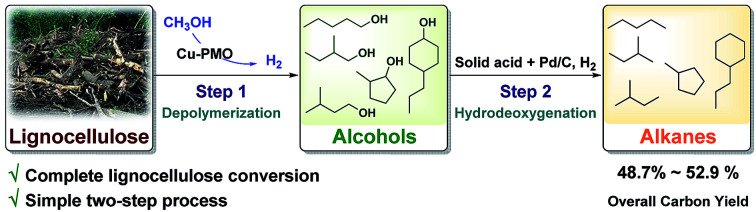

## Introduction

With the decreasing oil reserves and concerns related to global warming due to CO_2_ emissions, it is vital to explore more sustainable sources of energy, including renewables, for producing liquid transportation fuels.^1,2^ The conversion of raw biomass to liquid transportation fuels would mean the acquisition of a fossil fuel replacement that is both CO_2_ neutral and renewable as it can be extracted from plant material.^3,4^ Lignocellulose, which is already produced in large quantities (about 368 million dry tons annually could be produced on forestlands in the US alone)^5^ would be a starting material for the production of transportation fuels.^6^ Indeed, a variety of research directions have been established in the past few decades to attain this goal.^7^ Prominent research directions include thermal methods such as gasification (typically 600–1200 °C)^8^ or pyrolysis (typically 450–800 °C)^9^ which require higher temperatures, or liquid phase approaches that generally involve the fractionation (pretreatment) of lignocellulose^10^ to its main constituents – cellulose, hemicellulose or lignin and further conversion of these fractions. Important developments have been achieved in the production of second generation bioethanol,^11,12^ bio-butanol^13^ as well as alternative energy carriers such as GVL,^14–16^ levulinates^17,18^ or furanics^19,20^ obtained from cellulose or hemicellulose derived platform chemicals.

Producing liquid alkanes specifically has the advantage of delivering already known products, and such drop-in alkanes would be easily implemented into existing infrastructures.^21^ Indeed, several novel methods have been recently investigated regarding the conversion of lignocellulose to alkanes, most of which preferentially focused on the selective catalytic conversion of either lignin itself or cellulose derived platform chemicals. For example, organosolv lignin was converted to cycloalkanes in one-pot with high selectivity (>99%) by supported Ni catalysts^22,23^ or Ru-based bifunctional catalysts^24^*via* a cascade HDO reaction sequence. Liquid straight-chain alkanes were obtained directly from cellulose with high yield (up to 82%) in a biphasic catalytic system using tungstosilicic acid and Ru/C catalyst at 220 °C.^25^ Such systems have been also integrated into existing refinery structures.^26^ Regarding the (hemi)cellulose platform, several elegant routes have been developed for the transformation of single compounds HMF,^27,28^ furfural^29,30^ or the derived cyclopentanone^31^ into long chain alkanes that are suitable to use as jet fuels.^32^ These selective catalytic transformations generally comprise an acid or base catalysed aldol condensation or coupling, followed by deep HDO. Novel dehydrogenation/aldol condensation/hydrogenation sequences have also been reported for the conversion of the ABE fermentation mixtures to long chain ketones followed by deep HDO.^33,34^ Very recently we have developed a method for the coupling of mixtures of aliphatic alcohols obtained from the ‘Ligno-Flex’ process using a Cu and Ni doped porous metal oxide catalyst.^35–37^

Approaches that use raw lignocellulose directly may circumvent the relatively energy-, time- and material-intensive lignocellulose pretreatment/fractionation process, albeit the difficulty of product separation moves downstream. Recently, several elegant examples of direct conversion of lignocellulose to alkanes have been reported. Wang and co-workers described the direct HDO of raw lignocellulose sources into liquid alkanes over a multifunctional Pt/NbOPO_4_ catalyst.^38^ Ma and co-workers reported the efficient conversion of lignocellulose into gasoline alkanes (hexanes and pentanes), monophenols and related cycloalkanes over layered LiTaMoO_6_ and Ru/C in aqueous phosphoric acid medium.^39^

Copper doped porous metal oxides (Cu-PMOs) derived from synthetic hydrotalcites (HTC) have been established by Ford and co-workers as a highly interesting catalyst class for the conversion of renewable resources in supercritical methanol.^40–43^ Supercritical methanol, as well as the *in situ* generated hydrogen (from reforming a portion of the solvent) is beneficial for achieving hydrothermal conditions for the full conversion of lignocellulose solids into methanol soluble liquids under selected reaction conditions.^40,43^ The main products of the liquid phase consist of a predominantly C_2_–C_6_ range aliphatic alcohols from cellulose and C_9_-range, cyclic aliphatic alcohols from lignin and their methylated derivatives as methanol solution.^40,43^ Inspired by this precedence, we have attempted the detailed composition analysis of these mixtures, and investigate their conversion to more uniform mixtures of alkanes. First, we attempted to study the effect of different Cu-PMO catalyst compositions on the product distribution and the average chain lengths of the obtained alcohols. Second, we aimed to find ideal novel catalytic methods capable of deep HDO of such complex mixtures of predominantly aliphatic alcohols to liquid alkanes that would possess higher heating value than the corresponding oxygenates.^44^ Such a system has not yet been previously studied, very likely due to the methanol solvent, which may significantly complicate such downstream processing efforts.^36^ Herein we describe the successful, two-step conversion of lignocellulose to mixtures of alkanes. In addition, we have evaluated the reactivity of organosolv lignin extracted from pine lignocellulose as well as cellulose separately in order to compare the product portfolio to the lignocellulose conversion runs. For the corresponding HDO reactions, model compounds were used for investigating reactivity and optimization of the catalytic systems before moving to the more complex, real lignocellulose derived mixtures.

## Material and methods

### General considerations

Pine lignocellulose was purchased from Bemap Houtmeel B. V., elemental content C: 42.74%; H: 6.19%; O: 46.42%. All reagents and solvents are used as received without further purification. ZrO_2_, Nb_2_O_5_, Nafion, Pd/C (5%) catalysts was purchased from Sigma-Aldrich. Zeolite ZSM-5 (Si : Al 30 : 1) was purchased from Alfa Asea, before use it was calcined in the oven at 500 °C for 5 hours. USY-600 was purchased from Zeolyst International.

### Preparation and characterization of Cu-PMO catalysts

The HTC (hydrotalcite) catalyst precursor was prepared by a typical co-precipitation method.^45^ In a typical procedure, a solution containing AlCl_3_·6H_2_O (12.07 g, 0.05 mol), Cu(NO_3_)_2_·2.5H_2_O (6.98 g, 0.03 mol) and MgCl_2_·6H_2_O (24.40 g, 0.12 mol) in deionized water (0.2 L) was added to a solution containing Na_2_CO_3_ (5.30 g, 0.05 mol) in water (0.3 L) at 60 °C under vigorous stirring. The pH was kept between 9 and 10 by addition of small portions of a 1 M solution of NaOH. The mixture was vigorously stirred at 60 °C for 72 h. After cooling to room temperature, the light blue solid was filtered and re-suspended in a 2 M solution of Na_2_CO_3_ (0.3 L) and stirred for overnight at 40 °C. The solids were filtered and washed with deionized water until chloride free. After drying the solid for 6 h at 100 °C, 15.07 g of the hydrotalcite (HTC) was obtained. 4 g of obtained hydrotalcite was then calcined at 460 °C for 24 h in air and yielded 2.5 g of Cu20-PMO catalyst.

The catalyst prepared in this procedure is a porous metal oxide (PMO), denoted as Cu20-PMO, which indicates that in a 3 : 1 Mg/Al hydrotalcite precursor 20 mol% of the Mg^2+^ ions were replaced with Cu^2+^ ions. Other catalysts with different Cu content were prepared in the same procedure but using different amounts of Cu(NO_3_)_2_·2.5H_2_O and MgCl_2_·6H_2_O salt and named as Cu5-PMO and Cu10-PMO respectively.

Powder X-ray analysis was performed on a Bruker XRD diffractometer using Cu Kα radiation and the spectra were recorded in the 2*θ* angle range of 10°–90°. Elemental analysis was performed on a PerkinElmer instrument (Optima 7000DV).

### Extraction of lignin from pine lignocellulose

Extraction of lignin from pine lignocellulose was carried out in a 500 mL autoclave with an overhead stirrer and temperature controller. Typically the reactor was charged with 30 g of pine lignocellulose, 250 mL of methanol at room temperature. The reactor was sealed and stirred for 24 h at 170 °C. During the reaction 25 bar of autogenous pressure was developed. After completion of the extraction, the reactor was cooled down to room temperature. The mixture was collected in a 1000 mL beaker by rinsing the reactor several times with methanol and then filtered. The solids were washed with methanol and the combined solution was concentrated to 100 mL by rotary evaporator and precipitated with ice-cold water and then stirred overnight. The mixture was then centrifuged and the solids (Lignin) were collected by decanting the solutions which contained hemicellulose. The obtained solid was finally dried under vacuum yielding 0.66 g organosolv lignin.

### Catalytic conversion of pine lignocellulose (Step 1)

Pine sawdust (100 mg) and Cu-PMO catalysts (100 mg) were added to a 10 mL Swagelok stainless steel microreactor with 3 mL methanol as solvent. The reaction vessel was placed in a heating block at 320 °C for 6 h. The reactor was subsequently rapidly cooled down in an ice-water bath to terminate the reaction. The contents of the reaction vessel were transferred to a centrifuge tube and the solid was separated. The liquid layer was collected in a round bottom flask and the solid was washed twice with methanol. All liquid products were combined and methanol was removed by rotatory evaporation until approximately 5 mL liquid left. The products were then analyzed by GC-MS.

ATTENTION: Handle with care!! Upon heating to the indicated reaction temperature, autogenous pressure (>200 bar) develops in these vessels due to the supercritical methanol and *in situ* formed hydrogen.

### HDO of model compounds

Cyclohexanol (0.1 mL, 1 mmol) or alcohol mixture 1 (Mix1 containing 2-methylcyclohexanol, cyclohexanol, 4-methyl-2-pentanol, 2-methyl-1-butanol, 2-ethylhexanol, 2-cyclohexylethanol, 0.17 mmol for each and in total 0.128 mL) or alcohol mixture 2 (Mix2 containing 1-pentanol, 1-hexanol, 1-octanol, and 2-cyclohexylethanol, 0.25 mmol for each and in total 0.136 mL) were added to a reaction vessel with 50 mg solid acid catalyst and 10 mg Pd/C in 3 mL dodecane. The reaction vessel was placed in a high pressure stainless steel reactor (Endeavor©) and carefully purged with N_2_ for 3 times. Then the reactor was filled with H_2_ (10 bar) and heated to desired temperature. Next, the reaction mixture was transferred to a GC vial by filtration with a glass pipette equipped with a short pad of Celite and cotton pad. The products were analyzed by GC-MS and GC-FID.

### HDO of the product mixtures obtained by depolymerization of pine lignocellulose (Step 2)

#### Solvent exchange

Dodecane (5 mL) was added to the methanol solution obtained directly after lignocellulose depolymerization in Step 1. Then methanol was carefully evaporated by rotary evaporator to leave a dodecane solution behind which contains most of the products from Step 1. Small amount of precipitate (∼10 mg) was also observed, which was not further treated.

#### HDO

The dodecane solution obtained above was then transferred to reaction vessel containing 50 mg solid acid catalyst and 10 mg Pd/C. The reaction vessel was placed in a high pressure stainless steel reactor (Endeavor©) and purged with N_2_ for 3 times. Then the reactor was filled with H_2_ (10 bar) and heated to the desired temperature. The reaction mixture was next transferred to a GC vial by filtration over a PTFE filter and analyzed by GC-MS.

#### Upscaled HDO procedure

In order to more precisely calculate the final carbon yield and the yield of obtained alkanes, crude reaction mixtures from 10 separate small scale, 0.1 g pine lignocellulose depolymerization runs (see above Step 1) were combined. Then (10 mL) dodecane was added to this methanol solution and methanol was carefully evaporated by rotary evaporation resulting in a dodecane solution that contains most of the products from Step 1. The dodecane solution was then transferred to reaction vessel containing 0.5 g solid acid catalyst and 0.1 g Pd/C and subsequently, 10 mg eicosane was added as internal standard. The reaction vessel was placed in a 100 mL Parr reactor that was subsequently pressurized with 10 bar H_2_. After reaction was conducted for 4 hours at 180 °C, then the vessel was cooled to room temperature and depressurized. Next, the reaction mixture was transferred to a GC vial by filtration over a PTFE filter then analyzed by GC-MS and GC-FID. The gaseous products were collected into a gas bag and analyzed by GC-TCD.

### Analysis of liquid and gas products

Products of the liquid phase, obtained in Step 1 (alcohol mixtures) and Step 2 (alkanes) are analyzed by GC-MS and GC-FID. GC-MS was performed using a Shimadzu GC-2010 plus system equipped with a GCMS QP2010 GC SE detector and a HP-5 column. The column was maintained at 30 °C for 5 min, raised at 10 °C min^−1^ to 90 °C, then raised at 25 °C min^−1^ to 250 °C and maintained for 10 min. GC-FID was performed on a Hewlett Packard 6890 (HP-5 capillary column and a flame ionization detector (FID)). The column was maintained at 30 °C for 5 min, raised at 10 °C min^−1^ to 90 °C, then raised at 25 °C min^−1^ to 250 °C and maintained for 10 min. A fraction of short chain alkanes obtained in Step 2 are normally gaseous at room temperature. These were analyzed by GC-TCD upon collection in a gas bag. A HP5890 Series II GC equipped with a CP Porabond Q (50 m × 0.5 mm, film thickness 10 μm) and a CP-Molsieve 5A (25 m × 0.53 mm, film thickness 50 μm) column was used for this purpose. The injector temperature was set at 150 °C and the detector temperature at 90 °C. The oven temperature was kept at 40 °C for 2 min, then increased to 90 at 20 °C min^−1^, and kept at this temperature for 2 min.

### Calculation of the overall yield of obtained alkanes

The amount of obtained alkanes was calculated based on peak area in GC-FID with eicosane as internal standard.

Mass yield of obtained alkanes is calculated as follows:
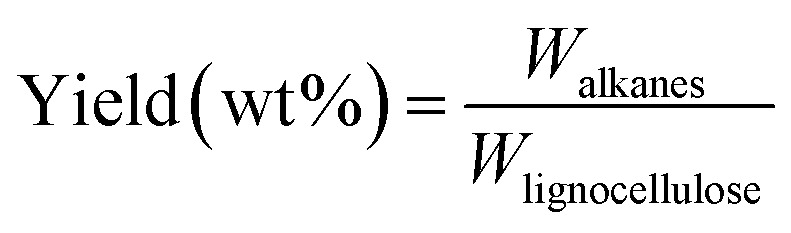
*W*_alkanes_ means the weight of total obtained alkanes, *W*_lignocellulose_ means the weight of total lignocellulose used in Step 1.
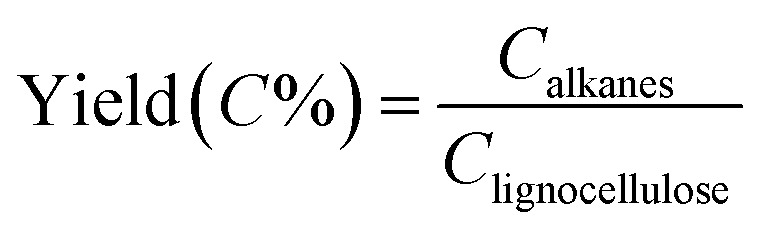
means the carbon content of total obtained alkanes, *C*_lignocellulose_ means the carbon content of total lignocellulose used in Step 1. The carbon content of the pine lignocellulose used in this study is 42.74%.

## Results and discussion

**Fig. 1:**
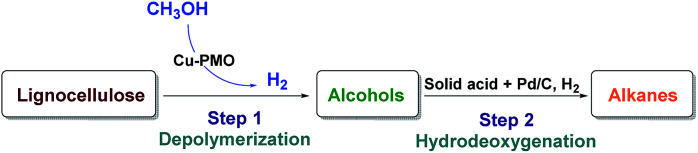
Two-step catalytic conversion of lignocellulose to alkanes.

With the aim of converting raw lignocellulose to alkanes we have set to investigate a specific depolymerization/HDO sequence shown in Fig. 1, which consists of the depolymerization of raw lignocellulose to alcohols (Step 1) using a Cu-PMO catalyst of varying composition in supercritical methanol and finding an appropriate method for the HDO of the produced alcohols to alkanes (Step 2).

### Depolymerization of lignocellulose with Cu-PMO catalysts

In order to investigate the influence of Cu content on reactivity, three different (HTC) precursors were prepared by co-precipitation, gradually replacing 5 mol%, 10 mol% and 20 mol% of Mg^2+^ by Cu^2+^ while keeping a constant 3 : 1 ratio between divalent and trivalent metal ions. The porous metal oxide catalysts were next obtained upon calcination of the HTC precursors at 460 °C for 24 hours.

The composition of HTC (Table S1†) were in good agreement with the theoretically expected values, indicating good incorporation of the metal ions. The formation of the double-layered HTC structure was confirmed by powder XRD measurements (Fig. S1a†). After calcination, the HTC structure was transformed into an amorphous mixed-oxide composition (Fig. S1b†) where the broad peaks at 37°, 43° and at 63° indicate the formation of spinels MgAlO_2_ and CuAlO_2_.

**Fig. 2:**
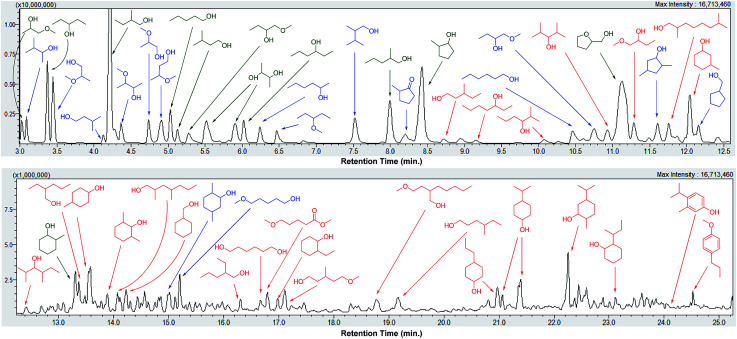
GC-MS trace of a product mixture obtained upon depolymerization of pine lignocellulose using Cu20-PMO in sc-MeOH. (Product labels: green = confirmed by the use of an authentic standard, blue = similarity index above 90%, red = similarity index between 70% and 90%.)

Depolymerization of pine lignocellulose in supercritical methanol (sc-MeOH) was carried out based on procedures described previously.^43^ After reaction, the transparent methanol solution of organic compounds was subjected to a detailed GC-MS identification, whereby the major products were unambiguously identified by the use of authentic standards, and the remaining signals were assigned based on GC-MS library search with a similarity index above 70%. A GC-MS trace of the sample after depolymerization of pine lignocellulose over Cu20-PMO, labeled with the chemical structures are shown in Fig. 2. Detail product analysis by different catalysts were listed in Tables S2 and S3.† As shown here, most of products are alcohols or hydroxyl ethers with small amount of aromatics also detected. The formation of hydroxyl ethers is attributed to the further reaction of methanol with formed alcohols.

**Fig. 3:**
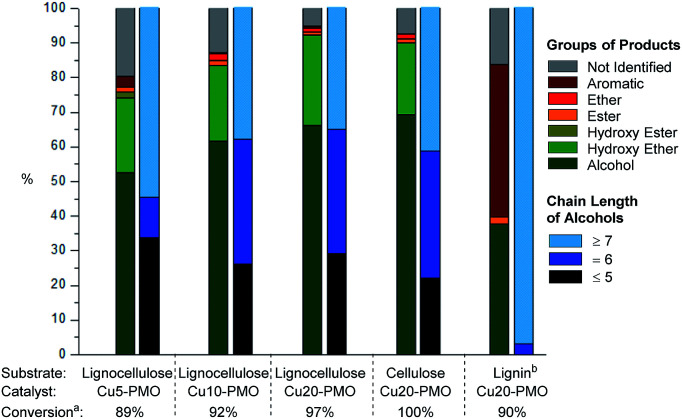
Conversion, product distribution and average chain length of alcohols in various product mixtures obtained upon catalytic depolymerization of different substrates in sc-MeOH over various PMO catalysts. Reaction conditions: substrate 100 mg, catalyst 100 mg, methanol 3 mL, 320 °C, 6 h. (a) Conversion is determined by gravimetric analysis. (b) Lignin was obtained from pine lignocellulose.

Upon careful analysis of the product mixtures obtained by using catalysts of varying Cu content, the results of products distributions as function of catalyst composition was summarized in Fig. 3 and Table S4,† which clearly shows that the conversion of raw lignocellulose (from 89% to 97%) and selectivity of alcohols (from 52.5% to 66.1%) increased with the increase of copper content. This is very likely due to different extent of methanol reforming leading to different hydrogen pressure, and therefore different extent of lignocellulose deconstruction. At the same time, products with chain length of more than 7 decreased slightly with the increase of copper content – this is also attributed to hydrogenolysis processes being more pronounced with increasing hydrogen pressure in the case of Cu20-PMO as expected, while Cu5-PMO should resemble more to a classical hydrotalcite, preferably promoting aldol condensation steps that lead to chain elongation.^33^

Organosolv lignin, separately obtained by lignocellulose fractionation, as well as pure cellulose were also reacted under the same reaction conditions with Cu20-PMO catalyst to evaluate the products obtained from these starting materials, serving as appropriate control experiments. When pure cellulose was used as starting material, the generated product mixtures had very similar distributions in the early retention time range, to those obtained from pine lignocellulose (Fig. 3) resulting in aliphatic alcohols as main products with 69.3% selectivity. Depolymerization of organosolv lignin gave good conversion of 90% and a product mixture mainly containing of substituted cyclohexanols and various phenolics (Fig. 3).

In summary, even though the mixture of products obtained with Cu5-PMO displayed slightly higher average chain length, the use of Cu20-PMO was most beneficial for achieving nearly full lignocellulose conversion (97% *vs.* 89% for Cu5-PMO). Therefore further experiments focused on the use of this catalyst composition. It has to be pointed out, that more research should focus on improving substrate/catalyst ratio for industrial application. The development of well-defined Cu nanoparticles or Cu containing bimetallic particles on appropriate supports would be desired.

### HDO of aliphatic alcohol model compounds

Since the product mixtures obtained upon catalytic conversion of pine lignocellulose in sc-MeOH using Cu20-PMO consisted of mainly aliphatic alcohols, establishing a suitable HDO procedure seemed feasible. Indeed, several catalytic systems, including the use of bifunctional catalysts^46,47^ or the combination of a solid acid and hydrogenation catalyst^48,49^ have been reported for HDO of aliphatic alcohols and phenolics to alkanes. In our case, the large excess of methanol and the complexity of product mixtures and possible etherification reactions are expected to be the major challenge. Therefore, first, suitable model compound mixtures were used to discern optimal catalytic conditions and catalyst type for the desired transformation and maximize the yield of alkanes. Inspired by systems that have previously shown high efficiency for HDO of lignin derived phenolics,^48,49^ we have selected to evaluate different solid acids (*e.g.* Nafion, HZSM-5, USY-600, ZrO_2_ and Nb_2_O_5_) in combination with Pd/C as preferred hydrogenation catalyst, in a broader temperature range (140–180 °C).

**Fig. 4:**
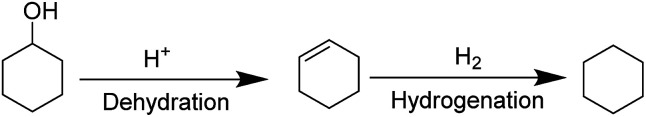
Reaction sequence in the HDO of model compound cyclohexanol.

The one pot alcohol to alkane conversion consists of the reaction sequence shown on Fig. 4, which starts with an acid catalyzed dehydration to yield the corresponding olefin, followed by a C=C hydrogenation by Pd/C. For establishing suitable reaction conditions, cyclohexanol was identified as suitable model substrate, and its reactivity was studied in the presence of different solid acids in combination with 10 mg Pd/C under the same conditions (140 °C, 4 h, 10 bar H_2_). As can be seen in Table 1, the two zeolites (HZSM-5 and USY-600) as well as Nafion were found to be promising candidates for the conversion of cyclohexanol, giving full substrate conversion at 140 °C after 4 hours.

**Table 1:** Conversion of cyclohexanol using different solid acid catalysts with Pd/C^a^

Entry	Solid acid	Conversion%	Selectivity%	
			Cyclohexane	Methylcyclopentane
1	HZSM-5	99	93	6
2	USY-600	99	100	0
3	ZrO_2_	8	100	0
4	Nb_2_O_5_	0	0	0
5	Nafion	99	99	1

a Reaction condition: 0.1 mL (1 mmol) cyclohexanol, 50 mg solid acid, 10 mg Pd/C, 140 °C, 4 h, 10 bar H_2_.

**Fig. 5:**
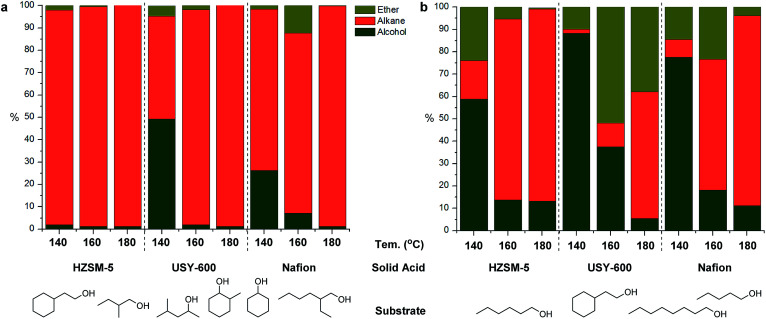
Product distribution upon conversion of alcohol model compounds ((a) Mix1 and (b) Mix2) using different solid acid catalysts. Reaction conditions: 0.128 mL alcohol mixture 1 or 0.138 mL alcohol mix 2, 50 mg solid acid catalyst, 10 mg Pd/C, 4 h, 10 bar H_2_.

Moving toward real lignocellulose-derived mixtures that contain both secondary and primary alcohols of varying chain length and complexity, we first prepared model mixtures Mix1 and Mix2 consisting of compounds relevant for the real lignocellulose depolymerization mixture. Mix1 consisted of equimolar amount of primary and secondary alcohols whereas Mix2 consisted of several primary alcohols (see Experimental section for detailed composition). Clear differences were seen in the reactivity of these mixtures (Fig. 5a*vs.*Fig. 5b) and especially for Mix1, the individual compounds have generally shown different reactivity pattern as well, depending on the catalyst used. While HZSM-5 was highly efficient leading to almost full conversion of all components of Mix1 at 140 °C, the other two solid acids tested showed significantly lower conversion at 140 °C, especially for primary alcohols (Fig. 5a). Increasing the reaction temperature further to 160 °C and 180 °C lead to full conversion of all alcohols and in several cases formation of ethers was also seen. To further investigate the formation of ethers and the reactivity of primary alcohols, Mix2 containing only primary alcohols was tested (Fig. 5b). As shown in Fig. 5b, generally poor reactivity was seen at 140 °C followed by a marked increase in primary alcohol conversion at 160 and 180 °C. While the substrate conversion increased dramatically for all substrate mixtures, product composition varied depending on the catalyst used in the case of Mix2. While HZSM-5 and Nafion gave almost the same results at 180 °C, and the yield of alcohols accounted for 86% and 85% respectively, with USY-600 as solid acid, substantial amount of ether by-product was found.

### HDO of alcohol mixtures from depolymerization of lignocellulose

**Fig. 6:**
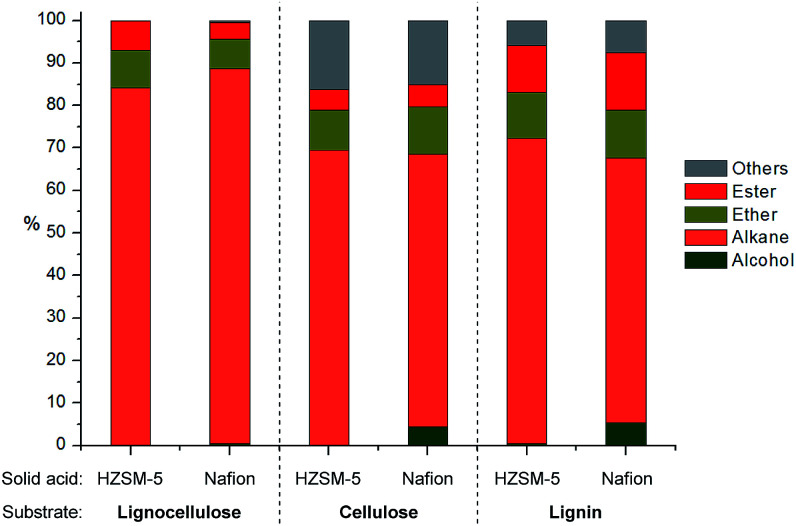
Distribution of products after HDO of mixtures obtained upon pine lignocellulose, cellulose or pine organosolv lignin depolymerization. Reaction conditions: 3 mL products mixture in dodecane, 50 mg solid acid catalyst, 10 mg Pd/C, 180 °C, 4 h, 10 bar H_2_.

Since both HZSM-5 and Nafion both performed well with model mixtures at 180 °C, they were both selected for the following tests for the upgrading of lignocellulose derived products to alkanes. The products from depolymerization of pine lignocellulose, cellulose and pine organosolv lignin with Cu20-PMO were used as starting materials. Since the presence of methanol would hamper reactivity in further HDO reactions, we first have developed a simple solvent exchange method, that consists of adding dodecane and careful removal of methanol through rotary evaporation, whereby the vast majority of compounds remained soluble. The dodecane solution, containing most of the products, was then used for the further upgrading to alkanes. As can be seen in Fig. 6, the product mixture from pine lignocellulose was converted to alkanes in high selectivity (>83%) with both HZSM-5 and Nafion as solid acid catalyst at 180 °C while ethers and esters remained unconverted under these reaction conditions. Slightly more of the latter two compound categories as well as other by-products (∼30%) were formed when using cellulose and pine organosolv lignin derived product mixture as substrate. The by-products are mainly ethers likely formed by etherification of the produced alcohols and diols. In the case of organosolv lignin, aromatic monomers or dimers may have also decreased catalyst activity for further HDO reactions. The higher alkane yield when using lignocellulose may be also attributed to the formation of formic acid from hemicellulose, as formic acid may act as good hydrogen donor for biomass conversion, facilitating the HDO process.^50^

**Fig. 7:**
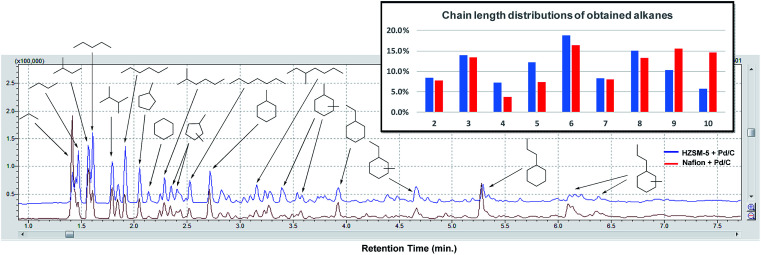
GC-MS trace of the obtained alkanes after HDO of alcohols obtained by pine lignocellulose depolymerization. Distribution of chain lengths of the obtained alkane products with HZSM-5 + Pd/C *versus* Nafion + Pd/C.

Next, the in-depth quantitative and qualitative analysis of the alkane product mixtures was performed using GC-MS, GC-FID and GC-TCD methods. In order to calculate the yield of the obtained alkanes more accurately, the HDO method was upscaled using a crude mixtures from depolymerization of 1 g pine lignocellulose. As shown in Fig. 7, the obtained alkanes reflect the general structure of lignocellulose: acyclic straight or branched alkanes mainly originate from cellulose or hemicellulose while most cycloalkane components are predominantly lignin-derived. Furthermore, the chain length of the obtained alkanes range from 2 to 10, while the collected gas samples contained mainly ethane and propane. Using Nafion as solid acid catalyst resulted in higher selectivity (30.3% *vs.* 16.2%) of long chain alkanes (>8) as shown in Fig. 7. This could be explained with the pore size differences between Nafion (>10 nm) and HZSM-5 (<1 nm).^51^ Furthermore, since HZSM-5 is also a good cracking catalyst,^52^ it may have facilitated the conversion of longer alkanes to shorter chain analogues. As shown in Table 2, the yield of the obtained alkanes using the Pd/C + Nafion reached 25.6 wt%, which is slightly higher than that obtained with the Pd/C + HZSM-5 catalyst system. The total carbon yield of the obtained alkanes was 48.7% and 52.9% respectively. A comparison of the alkane yield with other related research is summarized in Table S5.†

**Table 2:** Quantification of the obtained alkanes after Step 1 + Step 2 from 1 g pine lignocellulose

Catalysts for HDO	Weight of obtained alkanes (mg)										Yield	
	C_2_	C_3_	C_4_	C_5_	C_6_	C_7_	C_8_	C_9_	C_10_	Total	Wt%	C%
Pd/C + HZSM-5	22	28	18	31	47	20	37	26	14	192.7	19.3	48.7
Pd/C + Nafion	14	31	10	20	44	22	35	42	39	257.5	25.6	52.9

Possible reasons for loss of mass balance in this two-step process are: (a) hydrogenolysis of the lignin-methoxy groups would lead to gaseous products (*e.g.* methane) or methanol during Step 1; (b) loss of highly volatile organics during opening of Swagelok units as well as the solvent transfer procedure; (c) potential loss of compounds during solvent exchange (d) partial loss of highly volatile alkanes (<C_4_) obtained in Step 2 before analysis. In order to address these points, more elaborate equipment that allows for careful collection of each fraction and upscaling of these reactions will be required for future studies. Furthermore, testing of the combustion and fuel properties of these mixtures, which beneficially contain small amounts of methyl-ethers, as well as good amount of cyclic alkanes, should be determined in order to further asses their potential as bio-fuel alternatives.

## Conclusions

In summary, we have established a two-step catalytic procedure for the conversion of pine lignocellulose to predominantly a mixture of alkanes that reflect the composition of the original lignocellulose substrate. The first step consists of lignocellulose deconstruction in the presence of Cu-PMO catalysts. The second step involves the efficient HDO of these complex mixtures to linear and branched acyclic and cyclic alkanes, using Nafion and HZSM-5 in combination with Pd/C. Challenges related to the complexity of lignocellulose as well as the depolymerization mixtures have been overcome by in depth analysis, model compound and model mixture studies as well as proper quantification of the obtained alkane products. Gratifyingly, the aliphatic alcohol mixture obtained upon complete depolymerization of pine lignocellulose in Step 1 was fully converted to alkanes with an overall carbon yield around 50% in Step 2.

## Conflicts of interest

There are no conflicts to declare.

## Supplementary Material

RA-009-C9RA03174J-s001
