# Supplementary material for: Two-step catalytic conversion of lignocellulose to alkanes
Source: RSC Adv. 2019 Jul 30;9(41):23727–34. doi: 10.1039/c9ra03174j (PMC9069466; doi:10.1039/c9ra03174j)
Supplement: RA-009-C9RA03174J-s001 [file RA-009-C9RA03174J-s001.pdf]

Electronic Supplementary Material (ESI) for RSC Advances.
This journal is © The Royal Society of Chemistry 2019

Supporting information

Two-step catalytic conversion of lignocellulose to alkanes

Zhuohua Sun*, Daniel Buwalda, Katalin Barta*

Stratingh Institute for Chemistry, University of Groningen, Nijenborgh 4, 9747 AG Groningen, The Netherlands

***Corresponding author: Zhuohua Sun, Katalin Barta**

E-mail: sunzhuohua@yahoo.com ; k.barta@rug.nl

Phone: +31 50 36 34232

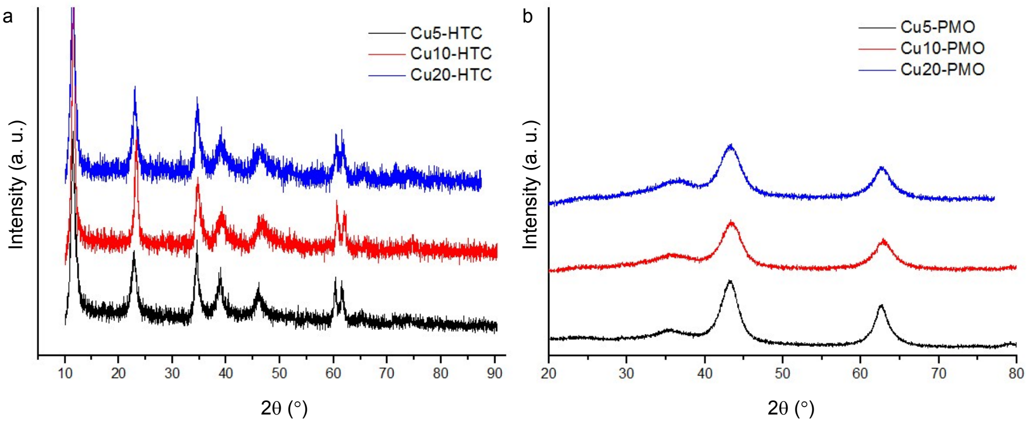

Figure S1 XRD patterns of the synthesized hydrotalcites (a) and the corresponding porous metal oxides (PMOs) after calcination (b).

Table S1 Elemental composition of prepared Cu-PMO catalysts.

Catalyst	Element content (wt %)			Theoretical composition	Experimental composition
	Cu	Mg	Al		
Cu5-PMO	4.4	31.15	12.4	$Cu_{0.15}Mg_{2.85}Al_1$	$Cu_{0.15}Mg_{2.79}Al_1$
Cu10-PMO	8.45	27.85	11.8	$Cu_{0.30}Mg_{2.70}Al_1$	$Cu_{0.30}Mg_{2.62}Al_1$
Cu20-PMO	16.25	24.35	11.35	$Cu_{0.60}Mg_{2.40}Al_1$	$Cu_{0.61}Mg_{2.38}Al_1$

Table S2 The composition of liquid products after reaction with lignocellulose or cellulose over different Cu-PMO catalysts.

Peak	Retention Time (Minutes)	Name	Lignocellulose			Celloluse
			Cu20-PMO	Cu10-PMO	Cu5-PMO	Cu20-PMO
1	3.03	2-Propanol, 1-methoxy-	0.9%	0.6%	0.1%	0.3%
2	3.09	2-Butanol, 3-methyl-,	0.9%	1.9%	0.1%	1.0%
3	3.37	3-Pentanol, 2-methyl-	3.8%	3.5%	0.4%	3.9%
4	3.45	1-Propanol, 2-methoxy-	3.1%	3.1%	1.1%	1.8%
5	3.58	Acetoin	0.0%	0.0%	0.4%	0.0%
6	4.12	1-Butanol, 3-methyl-	0.4%	0.4%	0.2%	0.0%
7	4.22	1-Butanol, 2-methyl-	11.6%	12.3%	4.3%	11.5%
8	4.37	2-Butanol, 3-methoxy-	0.9%	0.8%	0.2%	0.8%
9	4.74	1-Propanol, 2-methoxy-	1.4%	1.2%	0.4%	1.4%
10	4.91	1-Butanol, 3-methoxy-	1.9%	0.0%	3.5%	0.6%
11	5.03	1-Pentanol	1.9%	1.5%	0.2%	1.6%
12	5.13	3-Pentanol, 2-methyl-	0.7%	1.2%	0.1%	1.1%
13	5.28	2-Butanol, 1-methoxy-	1.0%	0.7%	0.3%	0.7%
14	5.52	2,3-Butanediol,	2.5%	0.0%	5.8%	0.0%
15	5.91	2,3-Butanediol,	1.5%	0.0%	5.9%	0.6%
16	6.02	3-Hexanol	1.3%	1.5%	0.0%	2.0%
17	6.24	2-Hexanol	1.1%	0.8%	0.3%	1.3%
18	6.47	Pentane, 3-methoxy-	1.0%	1.2%	0.2%	0.0%
19	7.52	1-Butanol, 2,3-dimethyl-	2.4%	3.2%	0.8%	2.9%
20	7.83	Butanoic acid, 2-hydroxy-, methyl ester	0.0%	0.0%	1.8%	0.0%
21	8.00	1-Pentanol, 2-methyl-	3.9%	4.4%	0.6%	4.1%
22	8.20	Cyclopentanone, 2-methyl-	0.7%	0.9%	0.4%	0.9%
23	8.42	Cyclopentanol, 2-methyl-, cis-	8.2%	4.7%	2.6%	6.6%
24	8.53	1,2-Butanediol	0.0%	0.0%	0.3%	0.0%
25	8.72	1-Pentanol, 3-methyl-	0.3%	0.4%	0.9%	0.6%
26	9.15	3-Heptanol	0.2%	0.4%	0.0%	0.5%
27	10.08	3-Hexanol, 2-methyl-	0.4%	0.8%	5.2%	0.7%
28	10.46	1-Hexanol	1.0%	1.2%	0.1%	1.4%
29	10.75	2-Butanol, 1-methoxy-	1.3%	0.0%	7.1%	1.2%
30	10.93	3-Pentanol, 2,4-dimethyl-	1.0%	0.0%	2.1%	1.0%
31	11.12	Tetrahydro-2-furanylmethanol	10.3%	9.9%	2.2%	8.4%
32	11.29	2-Butanol, 1-methoxy-	1.3%	0.8%	4.8%	0.7%
33	11.61	2,4-Dimethylcyclopentanol	1.5%	1.4%	4.1%	3.2%
34	11.75	1-Octanol, 2,7-dimethyl-	1.0%	0.8%	0.4%	0.0%
35	11.90	N. I.	0.0%	0.6%	0.3%	1.6%
36	12.04	Cyclohexanol, 3-methyl-	2.5%	4.9%	2.3%	4.7%
37	12.16	Cyclopentanemethanol	0.7%	1.1%	0.1%	1.0%
38	12.40	N. I.	0.0%	0.4%	0.1%	0.0%
39	12.69	3-Hexanol, 2,4-dimethyl-	0.4%	0.7%	1.0%	0.6%
40	13.10	N. I.	0.0%	0.5%	3.0%	0.0%
41	13.20	N. I.	0.0%	0.4%	0.5%	0.0%
42	13.31	Cyclohexanol, 2-methyl-	1.6%	3.4%	0.0%	4.5%
43	13.37	1-Pentanol, 3-ethyl-	0.6%	0.8%	2.5%	2.7%
44	13.56	Cyclohexanol, 4-methyl-	1.4%	2.1%	1.9%	3.1%
45	13.78	Cyclohexanol, 2,6-dimethyl-	0.3%	0.4%	0.3%	0.6%
46	13.89	3-Hexanol, 2,2-dimethyl-	0.5%	0.4%	2.1%	0.9%
47	13.99	1-Propene, 3-methoxy-2-methyl-	0.1%	0.8%	0.0%	1.5%
48	14.08	1-Heptanol, 2,4-dimethyl-	0.5%	0.0%	0.0%	0.7%
49	14.13	N. I.	0.0%	0.6%	1.0%	1.3%
50	14.23	Cyclohexanemethanol	1.3%	1.1%	0.5%	1.2%

51	14.31	N. I.	0.4%	0.0%	1.7%	0.7%
52	14.44	N. I.	0.7%	0.0%	0.0%	0.0%
53	14.57	N. I.	0.7%	0.4%	1.5%	1.8%
54	14.84	N. I.	0.0%	0.4%	0.6%	0.0%
55	14.88	N. I.	0.0%	0.4%	0.6%	0.0%
56	14.99	N. I.	0.9%	0.9%	0.9%	0.0%
57	15.02	Cyclohexanol, 2,4-dimethyl-	0.5%	0.4%	0.0%	1.4%
58	15.21	1-Pentanol, 5-methoxy-	1.7%	1.7%	0.0%	3.5%
59	15.57	N. I.	0.0%	0.5%	1.6%	0.0%
60	15.92	N. I.	0.0%	4.7%	0.4%	0.0%
61	16.31	3-Pentanol, 2,4-dimethyl-	0.6%	0.8%	1.9%	1.2%
62	16.24	N. I.	0.0%	0.0%	0.7%	0.6%
63	16.34	N. I.	0.0%	0.0%	1.0%	1.3%
64	16.67	1,5-Pentanediol	0.6%	0.0%	0.4%	0.5%
65	16.80	Pentanoic acid, 5-methoxy-, methyl ester	0.9%	1.4%	1.2%	1.0%
66	16.99	2-Ethylcyclohexanol	0.6%	0.4%	0.0%	0.0%
67	17.11	3-Methyl-5-methoxy-1-pentanol	1.3%	1.6%	1.4%	1.0%
68	17.48	N. I.	0.0%	0.0%	0.7%	0.0%
69	17.87	N. I.	0.0%	0.0%	0.4%	0.0%
70	18.32	N. I.	0.0%	0.8%	0.6%	0.0%
71	18.78	1-Heptanol, 2-(2-methoxyethyl)	1.3%	1.5%	0.6%	0.4%
72	19.17	1-Hexanol, 4-methyl-,	1.1%	0.0%	0.4%	1.0%
73	19.83	N. I.	0.0%	0.6%	0.7%	0.0%
74	20.97	2-Propylcyclohexanol	1.4%	0.0%	0.5%	0.0%
75	21.06	Cyclohexanol, 4-(1-methylethyl)-	0.6%	0.8%	0.6%	0.0%
76	21.40	Cyclohexanol, 4-(1-methylethyl)-	1.4%	0.6%	0.3%	0.0%
77	21.74	N. I.	0.0%	0.0%	0.9%	0.0%
78	22.26	Cyclohexanol, 2-methyl-5-isopropyl	2.9%	1.9%	1.3%	0.0%
79	22.74	N. I.	0.0%	0.4%	0.4%	0.0%
80	23.11	Cyclohexanol, 2-(sec-butyl)	0.5%	0.5%	0.4%	0.0%
81	23.60	N. I.	0.8%	0.5%	0.3%	0.0%
82	24.04	3-Methyl-4-isopropylphenol	0.1%	0.0%	0.8%	0.0%
83	24.52	Benzene, 1-methoxy-4-propyl-	0.4%	0.0%	0.5%	0.0%
84	25.23	N. I.	0.0%	0.4%	0.8%	0.0%
85	25.42	N. I.	0.0%	0.4%	0.9%	0.0%
86	25.83	N. I.	0.9%	0.0%	0.3%	0.0%
87	25.81	N. I.	0.0%	0.0%	0.8%	0.0%
88	25.98	N. I.	0.0%	0.0%	0.7%	0.0%
89	26.38	N. I.	0.7%	0.0%	0.4%	0.0%

Table S3 The composition of liquid products after reaction with pinewood organosolv lignin over Cu20-PMO catalysts (area %).

Peak	Ret. Time (Min.)	Name	Area %	Peak	Ret. Time (Min.)	Name	Area %
1	11.16	Cyclohexanol	0.5%	37	24.06	3-Methyl-4-isopropylphenol	1.2%
2	12.19	Cyclopentanemethanol	0.7%	38	24.50	N. I.	1.3%
3	13.35	Cyclohexanol, 2-methyl-	0.7%	39	24.55	Benzene, 1-methoxy-4-propyl-	1.6%
4	13.60	Cyclohexanol, 3-methyl-	0.6%	40	24.62	Phenol, 4-(1-methylpropyl)-	1.0%
5	14.12	N. I.	1.4%	41	24.82	3-Methyl-4-isopropylphenol	1.8%
6	15.01	2,5-Dimethylcyclohexanol	1.2%	42	24.87	Benzenemethanol, 4-methoxy-	0.5%
7	15.05	Cyclohexanol, 2,4-dimethyl-	0.5%	43	24.99	Phenol, 2,3,5,6-tetramethyl-	3.0%
8	15.61	Cyclohexanol, 2,3-dimethyl-	0.4%	44	25.10	N. I.	0.5%
9	15.79	2,5-Dimethylcyclohexanol	0.5%	45	25.25	Benzene, 1-(1,3-dimethyl-3-butenyl)-4-methoxy-	2.6%
10	17.12	Phenol, 2-methyl-	0.4%	46	25.35	Cyclohexanone, 2-cyclohexylidene-	1.5%
11	17.32	Cyclohexanol, 5-methyl-2-(1-methylethyl)-	1.2%	47	25.46	3-Cyclohexene-1-propanol	2.2%
12	17.42	2-Ethylcyclopentylmethanol	1.7%	48	25.54	Naphthalene, 2-methyl-	7.1%
13	18.86	Cyclohexanol, 3-methyl-2-(1-methylethyl)-	1.0%	49	25.71	N. I.	0.6%
14	19.10	Phenol, 2,6-dimethyl-	0.5%	50	25.82	Thymol	4.9%
15	20.93	Phenol, 2,4-dimethyl-	1.5%	51	25.93	N. I.	1.5%
16	21.01	2-Propylcyclohexanol	1.2%	52	26.00	Cyclohexanol, 2-methyl-5-(1-methylethenyl)-	1.2%
17	21.10	Cyclohexanol, 4-(1-methylethyl)-	0.5%	53	26.03	N. I.	0.9%
18	21.39	Cyclooctanemethanol	2.2%	54	26.30	N. I.	0.8%
19	21.43	Cyclohexaneethanol	1.7%	55	26.41	N. I.	0.8%
20	21.58	Cyclohexanol, 3,3,5-trimethyl-	0.4%	56	26.55	Benzene, 1,2,4-trimethyl-5-(1-methylethyl)-	1.0%
21	21.73	3-Phenylpropanol	0.6%	57	26.59	N. I.	0.7%
22	21.93	3-Methyl-2-(3-methylpentyl)-3-buten-1-ol	0.8%	58	26.64	N. I.	0.8%
23	22.06	N. I.	0.8%	59	26.77	Phenol, 2-methyl-4-(1,1,3,3-tetramethylbutyl)-	4.6%
24	22.28	Cyclohexanol, 2-methyl-5-(1-methylethyl)-	2.6%	60	26.98	Phenol, 2-methyl-6-(2-propenyl)-	1.3%
25	22.41	Phenol, 2,3,6-trimethyl-	1.2%	61	27.13	N. I.	0.7%
26	22.51	Cyclohexanemethanol, 4-(1-methylethyl)-, cis-	1.9%	62	27.25	N. I.	0.9%
27	22.58	Cyclohexanemethanol, 4-(1-methylethyl)-, trans-	0.9%	63	27.44	N. I.	0.8%
28	22.62	Cyclohexanemethanol, 4-t-butyl-2-hydroxy-	0.9%	64	27.51	2,5-Diethylphenol	1.1%
29	22.76	2,2,5-Trimethyl-cyclohexane-1,4-diol	1.1%	65	27.68	3-Ethylphenol, methyl ether	1.8%
30	22.92	Cyclohexanol, 2-methyl-5-(1-methylethyl)-	0.7%	66	27.93	Benzene, 1-methoxy-4-(1-methylpropyl)-	1.4%
31	22.99	Phenol, 2,3,6-trimethyl-	1.2%	67	28.15	N. I.	1.0%
32	23.15	Benzenemethanol, 4-methoxy-	2.0%	68	29.56	N. I.	0.8%
33	23.49	Cyclohexanol, 3,3,5-trimethyl-	2.7%	69	29.87	N. I.	0.8%
34	23.63	Phenol, 2,4,5-trimethyl-	1.6%	70	30.92	n-Nonadecanol-1	2.3%
35	23.71	Cyclopentanol, 1,2-dimethyl-3-(1-methylethenyl)	1.9%	71	31.18	Methyl stearate	2.0%
36	23.87	Cyclohexanol, 2-methyl-3-(1-methylethenyl)-	2.4%	72	32.94	N. I.	0.9%

Table S4 Distribution of products after depolymerization reaction from different substrates over different catalysts.

Catalysts	Substrate	HET	AL	HES	ES	ET	AR	N. I.
Cu20-PMO	Lignocellulose	26.3%	66.1%	0.0%	0.9%	1.1%	0.5%	5.0%
Cu10-PMO	Lignocellulose	21.8%	61.6%	0.0%	1.4%	2.0%	0.4%	12.8%
Cu5-PMO	Lignocellulose	21.7%	52.5%	1.8%	1.2%	0.2%	3.0%	19.6%
Cu20-PMO	Cellulose	20.8%	69.3%	0.0%	1.0%	1.5%	0.0%	7.4%
Cu20-PMO	Lignin	0.0%	37.8%	0.0%	2.0%	0.0%	44.0%	16.2%

HET: Hydroxyl Ether; AL: Alcohol; HES: Hydroxyl Ester; ES: Ester; ET: Ether; AR: Aromatic; N. I.: Not Identified.

Table S5 Comparison of the process focusing on producing alkanes from lignocellulose.

Substrate	Catalyst	Temperature °C	Products	Yield %	Ref.
Corn stalk	$LiTaMoO_6$ and Ru/C	230	Gasoline alkanes	82.4	1
Birch	$Pt/NbOPO_4$	190	Pentanes<br>Hexanes<br>Alkylcyclohexanes	54.0<br>63.4<br>33.7	2
Pine	Step 1: Cu-PMO<br>Step 2: Pd/C+Nafion	Step 1: 320<br>Step 2: 180	C2-C10 alkanes	52.9	This work

Consumption of hydrogen

The desired hydrodeoxygenation proceeds via **Step 1** combined with **Step 2**. While in **Step 1**, the hydrogen equivalents originate from the solvent itself upon its reforming, in **Step 2**, externally introduced hydrogen gas was used. Assuming almost full hydrodeoxygenation to alkanes after (**Step 1** + **Step 2**), a simple calculation of the overall hydrogen demand can be presented based on the actual oxygen content of the lignocellulose used. The oxygen content in the pine lignocellulose used is 51.02%. 1 g of pine lignocellulose contains 0.51 g (31.9 mmol) oxygen. In total HDO, all the oxygen will be finally converted to water and will consume 31.9 mmol H_2 (0.0638 g).

References:

1. Y. Liu, L. Chen, T. Wang, Q. Zhang, C. Wang, J. Yan and L. Ma, *ACS Sustainable Chemistry and Engineering*, 2015, **3**, 1745–1755.
2. Q. Xia, Z. Chen, Y. Shao, X. Gong, H. Wang, X. Liu, S. F. Parker, X. Han, S. Yang and Y. Wang, *Nature Communications*, 2016, **7**, 11162.
